# The complete chloroplast genome sequence of *Gelidocalamus xunwuensis* (Bambusoideae: Arundinarieae): a shrubby bamboo endemic to China

**DOI:** 10.1080/23802359.2019.1673679

**Published:** 2019-10-04

**Authors:** Yi-Ting Zhang, Chun-Ce Guo, Guang-Yao Yang, Fen Yu, Wen-Gen Zhang

**Affiliations:** Jiangxi Provincial Key Laboratory for Bamboo Germplasm Resources and Utilization, Forestry College, Jiangxi Agricultural University, Nanchang, P. R. China

**Keywords:** Poaceae, *Gelidocalamus xunwuensis*, phylogeny, temperate woody bamboos

## Abstract

The complete chloroplast genome sequence of *Gelidocalamus xunwuensis*, firstly determined here, is 139,705 bp in length, inclusive of a pair of inverted repeat (IR, 21,817 bp) regions separated by a small single copy (SSC, 12,803 bp) and a large single copy (LSC, 83,268 bp). It contains 132 genes, such as 85 CDS, 8 rRNA genes, and 39 tRNA genes, respectively. The phylogenetic analysis shows that *G. xunwuensis* is highly clustered in the *shibataea* clade (III) of Arundinarieae, sister to the clade of *G. tessellatus + Ferrocalamus rimosivaginus*.

*Gelidocalamus xunwuensis* W. G. Zhang and G. Y. Yang is potentially ornamental for its graceful appearance (e.g. several branches per node and leaves usually solitary on each ultimate branch) in the family Poaceae (Bambusoideae: Arundinarieae) (Zhang et al. 2017). This species, endemic to southern China, may play an important role in understanding the distribution and evolution of the genus *Gelidocalamus*. However, the genetic data of *Gelidocalamus* are still scarce, and up to now only the chloroplast genome of *G. tessellatus* was completed (Ma et al. 2014). In this study, by the method of genome-skimming sequencing, the complete chloroplast genome of *G. xunwuensis* was firstly obtained, to accelerate the studies on the molecular phylogeny of *Gelidocalamus*.

Total genomic DNA was extracted from leaves of *G. xunwuensis* dried with silica gel, which was collected from the type locality Xunwu County of Jiangxi Province (24°54′1.59″N, 115°28′2.78″E). The voucher specimen (accession number 1107, JXAU!) was deposited at the herbarium of the College of Forestry, Jiangxi Agricultural University, China. Illumina paired-end (PE) library was prepared and sequenced in the Kunming Institute of Botany, Chinese Academy of Sciences (CAS) in Kunming, China. By using SPAdes 3.13.0 (Bankevich et al. 2012) and Geneious 9.0.5 (http://www.geneious.com/), all contigs of the chloroplast genome sequence were spliced and assembled. Then, the webserver DOGMA (Wyman et al. 2004) was applied to annotate the complete chloroplast genome and Simple sequence repeats (SSR) were detected by MISA (http://pgrc.ipk-gatersleben.de/misa).

The complete chloroplast genome of *G. xunwuensis* is 139,705 bp in length, inclusive of a typical quadripartite structure with two inverted repeats (IRs) of 21,817 bp separated by a large single copy (LSC) of 83,268 bp and a small single copy (SSC) of 12,803 bp. A total of 132 genes in the chloroplast genome, including 85 protein-coding genes, 8 ribosomal RNA (rRNA) genes and 39 transfer RNA (tRNA) genes, are identified. Nine protein-coding genes, such as *atpF*, *ndhA*, *ndhB*, *petB*, *petD*, *rpl16*, *rpl2*, *rps12*, and *rps16*, respectively, have one intron each and one gene (i.e. *ycf3*) contains two introns. Protein-coding regions (CDS) contribute 41.53% of the chloroplast genome and the total of GC content is 38.9%. In a word, the chloroplast genome of *G. xunwuensis* shows a highly conserved genome structure of temperate woody bamboos (Arundinarieae) (Zhang et al. 2011). Furthermore, 34 SSR sites are detected in the cp genome of *G. xunwuensis*.

To determine the phylogenetic status of *G. xunwuensis*, additional 30 complete chloroplast genomes of the trib. Arundinarieae, together with 3 species as outgroup (Figure 1), were downloaded from NCBI. By using RAxML 8.2.8 (Stamatakis 2014) and MrBayes 3.2.6 (Andres et al. 2013), the phylogenomic analysis of Arundinarieae was performed again. Results show that *G. xunwuensis* is highly clustered in the shibataea clade (III) of Arundinarieae, being sister to the clade of *G. tessellatus* + *Ferrocalamus rimosivaginus*. The phylogenetic relationship of 11 major lineages of Arundinarieae recovered here is congruent with recent studies (Ma et al. 2014; Zhang and Chen 2016). The internodes in the ML tree are short, indicating a probable recent rapid radiation of Arundinarieae.

**Figure 1.**
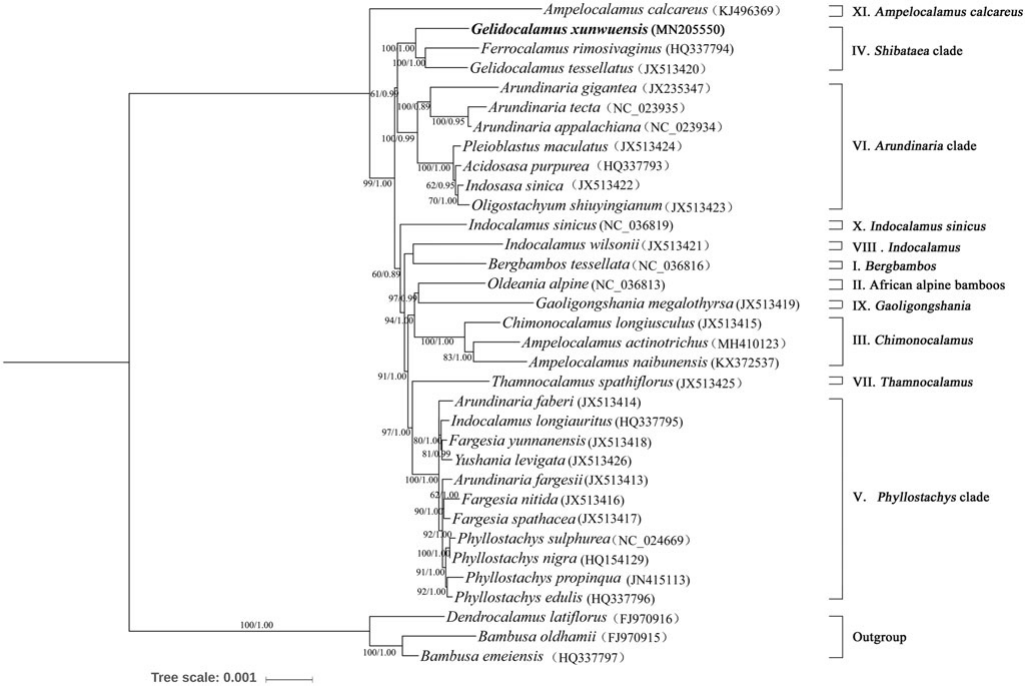
Phylogram of the 50% majority-rule consensus tree inferred from 34 woody bamboo chloroplast genomes. Numbers above branches indicated the maximum likelihood bootstrap support and the posterior probabilities, respectively.
